# Postural Control and Sensory Processing in Preschool Children with Autistic Spectrum Disorder: A Cross-Sectional Study

**DOI:** 10.3390/children11030303

**Published:** 2024-03-05

**Authors:** Marta Ferreiro-Pérez, Vanesa Abuín-Porras, Patricia Martín-Casas, Rosa M. Ortiz-Gutiérrez

**Affiliations:** 1 Centro de Atención Temprana de Parla (ADEMPA), 28981 Madrid, Spain; fisioterapia.04v@adempa.org; 2 Faculty of Sport Sciences, Universidad Europea de Madrid, 28670 Madrid, Spain; vanesa.abuin@universidadeuropea.es; 3 Department of Radiology, Rehabilitation and Physiotherapy, Faculty of Nursing, Physiotherapy and Podiatry, Complutense University of Madrid, 28040 Madrid, Spain; rosaorti@ucm.es; 4 The Health Research Institute of the Hospital Clínico San Carlos (IdISSC), 28040 Madrid, Spain

**Keywords:** Autistic Spectrum Disorder, pediatrics, balance, sensory processing, disability evaluation, technology assessment

## Abstract

The role of sensory processing in maintaining postural control (PC) among preschool-aged children with autism spectrum disorder (ASD) remains underexplored despite its potential implications for their developmental trajectory. This study aimed to assess the utilization of sensory information for PC maintenance while standing in preschool-aged children with ASD and to examine its correlation with PC during functional tasks using a standardized tool. The cross-sectional study recruited 27 children, aged between 3 and 6 years, diagnosed with ASD. Participation indexes for somatosensory, vestibular, visual, and visual preference were computed during a modified Clinical Test of Sensory Integration and Balance (m-CTSIB), based on sagittal plane body sway analyzed via video with Kinovea^®^ software (version 0.9.4). Additionally, scores from the Pediatric Balance Scale (PBS) were analyzed. Statistical analysis of data derived from lateral malleolus and mastoid process sway using the Friedman test revealed significant differences in the utilization of various sensory systems involved in PC during the m-CTSIB (*p* < 0.001). There was a pronounced reliance on somatosensory information, coupled with increased instability in the absence or with the variability of visual information. The mean PBS score was 50.44 ± 2.74, exhibiting a significant negative correlation with the vestibular index (*p* < 0.05). Preschool-aged children with ASD demonstrated challenges in maintaining PC while standing under different sensory conditions, indicating a heightened dependence on somatosensory cues, particularly in the absence or with the variability of visual stimuli. While these challenges were not reflected in PBS scores, they were negatively correlated with the vestibular index.

## 1. Introduction

Autism spectrum disorders (ASD) are classified among neurodevelopmental disorders, representing a prevalent condition with a high hereditary component [1], exhibiting a prevalence rate of 1 of in 36 children between the ages of 3 and 8 years old [2]. This ratio is influenced by gender, as it was 3.8 times more prevalent among boys than girls (43 versus 11.4) [2]. Although, traditionally, no significant differences have been described by ethnicity, culture, or sociodemographic factors [3], in the last study carried out in the United States, the prevalence of ASD was lower among white children than other racial and ethnic groups [2].

Some characteristics of ASD can be observed from an early age, with the most relevant being difficulties in communication and social interaction, alterations at the sensory level, impairment in intellectual capacity at various levels, and restrictive patterns in behavior [1,4]. Previous studies have described sensory alterations in children with ASD [5], as well as delays in gross and fine motor skills [6], particularly difficulty in motor coordination, characterized by clumsiness, postural instability [7], difficulties in reaching objects, and abnormal gait patterns [5]. Additionally, these difficulties are interrelated; for example, the lack of postural control (PC) has been associated with their social development, as they may find it challenging to engage in sports and leisure activities [8]. Concerns about early signs in ASD have recently increased; although deficits in social communication and interaction, as well as restricted interests and repetitive behaviors, are considered cardinal signs of ASD, delays of both gross and/or fine motor abilities have also been reported. Moreover, an impairment of multisensory integration has been described and implicated in the development of various other clinical features of ASD [9].

Regarding PC, children with ASD may exhibit standardized results in balance tests such as the Pediatric Balance Scale (PBS), potentially resulting in inadequate treatment tailored to their functional needs. However, balance disorders have a detrimental impact on their activities and participation. The limited detection of balance disorders at an early age may be attributed to the instruments used in their assessment, which typically do not encompass aspects related to the utilization of sensory information involved in maintain balance [8].

Furthermore, balance impairment persists into adulthood. Morris et al. evaluated individuals aged between 19 and 35 years with ASD using posturography and observed greater postural sway in this group compared to typically developing (TD) individuals across various standing tasks with different sensory and cognitive demands (e.g., standing with closed eyes, on an unstable platform, performing dual tasks, and engaging in a visual search) [10]. Understanding how children with ASD process and utilize sensory information to maintain postural control from childhood is crucial for providing evidence on sensorimotor processing development and detecting impairments that influence functional performance, thus enhancing therapeutic interventions [10,11].

In this regard, the Sensory Organization Test (SOT) is a specific posturography test regarded as the “gold standard” for studying PC [12]. This test assesses the interaction of visual, vestibular, and somatosensory inputs in maintaining balance against gravity. Studies examining the utilization of sensory inputs in balance maintenance in children with ASD based on posturography are limited. This is mainly due to the challenges of obtaining and adapting current laboratory systems, such as posturography, to the characteristics of these children, especially those less than 6 years old [13]. Indeed, some authors have employed instrumental measures to evaluate PC in children to explore how divergent patterns of sensory processing could account for differences between children with ASD and TD. However, such studies have primarily focused on children older than 6 years [13,14].

The Clinical Test of Sensory Interaction on Balance (CTSIB) was developed as a clinical adaptation of the SOT, monitoring the sway patterns of individuals as they endeavor to maintain PC while standing [15]. This test has been validated in the pediatric population by Gagnon et al. and termed the modified CTSIB (m-CTSIB). The m-CTSIB was devised to evaluate sensory contributions to balance, enabling the calculation of various sensory participation indexes representing the utilization of different sensory information to sustain PC during standing. The most employed sensory participation indexes include somatosensory, vestibular, and visual inputs, although other variations may be introduced depending on age and sample characteristics. To calculate these indexes, some research focuses on assessing performance in terms of sway (e.g., degrees/second) rather than the duration for which the subject maintains the initial position [16]. In recent years, the emergence of free computer programs designed for movement analysis using photogrammetry techniques (image and video) has presented an intriguing alternative, facilitating quantitative analysis of postural strategies required to maintain stability through observational tests. These computer programs enable objective evaluation of postural sway in various planes and reference points [17].

In contrast to the gold standard measurement obtained with posturography devices, video capture and movement analysis software such as Kinovea^®^ offer an accessible and cost-effective system for studying balance. Furthermore, different modifications such as the introduction of visual interferences can be easily incorporated into the assessment. This affords a closer approximation to the real balance capacity of these children. Additionally, since the analysis is based on recorded video images, this software enables repeated viewing of the test for more in-depth analysis, rendering it an ideal tool for studying PC in children with ASD [18].

Moreover, children with ASD often experience heightened anxiety when assessed in unfamiliar environments [19]. Therefore, as an innovative approach in our study, the utilization of video analysis systems enables the evaluation of balance within their familiar surroundings. This novel aspect promotes their acceptance and engagement in the assessment process, which holds particular significance for children under the age of 6.

In this study we investigated whether children with ASD utilize visual, vestibular, and somatosensory information differently to maintain PC while standing. This was assessed by examining sensory participation indexes during the m-CTSIB, measuring sagittal sway of the lateral malleolus and mastoid process. Additionally, we sought to determine whether difficulties in PC could be identified using a standardized measure, the PBS, and whether its results were correlated with sensory participation indexes from the m-CTSIB. Thus, the primary objective of this study was to evaluate whether there were differences in the utilization of sensory information to maintain PC while standing in preschool children with ASD. Furthermore, our secondary objectives were to assess the agreement between each sensory participation index calculated based on sway from the lateral malleolus and mastoid process during the m-CTSIB and to examine the relationship between the sensory participation indexes of the m-CTSIB and PC during functional tasks, as measured by the PBS.

## 2. Materials and Methods

### 2.1. Study Design

The research presented is a cross-sectional observational study, conducted in accordance with the criteria established by the Strengthening the Reporting of Observational Studies in Epidemiology [20]. Approval for the study was obtained from the Clinical Research Ethics Committee of Hospital Clínico San Carlos (internal code 22/093-E_TFM), and all procedures were conducted in compliance with the guidelines of the Declaration of Helsinki [21].

### 2.2. Subjects

The study included children between 3 and 6 years of age diagnosed with ASD, who were receiving services at an Early Intervention Center (ADEMPA). Prior to data collection, informed consent was obtained from the participants, parents, or guardians.

The sample size was determined based on data from a previous study [22] investigating motor deficits in children with an ASD of similar age to our sample. Using a confidence level of 95%, a margin of error of 5%, and an estimated deviation percentage of 50%, a sample size of 20 participants was initially proposed. However, to account for potential attrition, a final sample size of 30 participants was targeted.

A non-probabilistic convenience sampling approach was employed, with consecutive cases recruited until the desired sample size was reached. Participants were recruited from among children attending ADEMPA. Inclusion criteria comprised children with ASD aged 3 to 6 years who could stand upright independently. Exclusion criteria included a diagnosis of neurological, cardiorespiratory, or orthopedic conditions that could impair evaluation performance; difficulties comprehending and executing simple activities via verbal instructions and/or imitation; and pharmacological treatment that could affect the child’s motor function.

### 2.3. Outcome Measures

Initially, participant’s parents or guardians provided informed consent and consent for the collection of development milestone data from the children’s clinical records.

Subsequently, children completed balance assessment during a single session, with rest periods tailored to individual needs. Parents or guardians were requested to ensure children were dressed comfortably for assessments.

All outcome measures were conducted by the same researcher, a physiotherapist with extensive experience working with children with ASD (MFP). Prior to assessments, children’s heights and weights were recorded using a medical weigh scale with a height gauge (model SECA711).

#### 2.3.1. Modified Clinical Test of Sensory Integration and Balance (m-CTSIB)

We recorded the somatosensory, vestibular, and visual participation indexes, as well as the visual preference index, as outcome variables. These metrics align with those utilized in m-CTSIB, considered the gold standard for PC assessment [23]. Index calculations were based on anterior and posterior body swinging recordings during six balance conditions with varying sensory demands:

C1: Standing upright on a stable surface with eyes open.
C2: Standing upright on a stable surface with eyes closed.
C3: Standing upright on a stable surface with eyes open and visual interference.
C4: Standing upright on an unstable surface (foam rubber base of 60 × 40 × 5.5 cm) with eyes open.
C5: Standing on an unstable surface with eyes closed.
C6: Standing upright on an unstable surface with eyes open and visual interference.

Two conditions involving visual interference were added to the original version [16]. Visual interference was provided by presenting a video featuring alternating black and white abstract images and a completely white image, specially designed for children with visual impairments (See Supplementary Materials, Video S1: Visual interference).

Adhesive markers were placed on the lateral malleolus and mastoid process as reference points for recordings, as ankle reaction is the primary adjustment resource, and overall body roll needs to be considered [24,25,26]. Children were instructed to stand without shoes, with trousers above the ankles, ensuring clear visibility of the lateral malleolus without discomfort. The test was conducted on both a firm surface with foot position markers for foot position and a foam rubber base with the same markings. Children were required to maintain stable PC while standing for three consecutive 20 s trials in all test conditions. The first trial was disregarded as a familiarization trial, with the subsequent two trials averaged for analysis [13].

The sensory participation indexes were calculated using the following formulas [26]: The somatosensory index (SOM) ([C2/C1] × 100) evaluated the participant’s ability to utilize somatosensory input to maintain PC. The visual index (VIS) ([C4/C1] × 100) assessed the participant’s ability to utilize visual input for maintaining PC. The vestibular index (VEST) ([C5/C1] × 100) evaluated the participant’s ability to utilize vestibular input for maintaining PC. Additionally, the visual preference index (VP) ([C3 + C6/C2 + C5] × 100) assessed the extent to which the participant relied on visual information for maintaining PC while standing, even when this information is presented as interference due to its high variability [16]. Greater body swinging indicates greater instability and, consequently, less efficient utilization of the sensory information required to maintain PC in each condition. Therefore, higher index values indicate less utilization of that sensory input for maintaining PC while standing [13].

**Figure 1:**
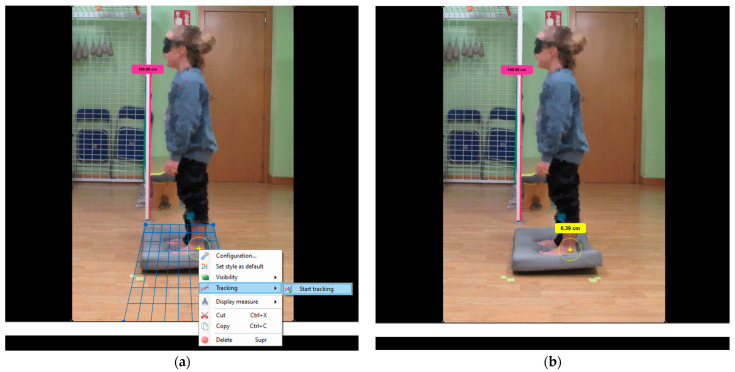
(**a**,**b**) Body sway recording and calculation of total body swinging in the standing upright on an unstable surface with eyes open condition (C4) from the lateral malleolus marker using Kinovea^®^ software.

To track body displacement, participants were recorded in the left sagittal plane using a Samsung NX3000 camera (Samsung Electro-Mechanics, Seoul, Republic of Korea) placed 1.5 m away and at a height of 50 cm relative to the participant, allowing visualization of the entire body while standing. Subsequently, the open-access software Kinovea^®^ Motion Analysis Software (GNU General Public License version 2) was utilized for video analysis [27]. The tracking displacement tool in Kinovea^®^ (version 0.9.4) was employed to estimate body displacements in centimeters across the different frames of the video (Figure 1a,b).

#### 2.3.2. Pediatric Balance Scale (PBS)

The PBS was developed to assess functional balance in the pediatric population based on modifications of the Berg Balance Scale. It consists of 14 items evaluating various functional activities that children can perform at home, at school, and in their environment, with a maximum score of 56. Administration of the scale followed these instructions: for items 1, 2, 3, 6, 7, and 8, the best of three attempts was recorded; for items 9 and 14, the mean of three attempts was recorded; and for items 4, 5, and 10–13, only one attempt was permitted [28].

### 2.4. Statistical Analysis

Since the variables analyzed displayed significant deviations from normal distribution, non-parametric tests were employed for all analyses.

The Friedman test was utilized to examine differences among scores of the VIS, VEST, SOM, and VP indexes calculated from m-CTSIB conditions and used in the sample of children with ADS to maintain PC. For further analysis, when significant differences were detected, the Durbin–Conover test was employed for post hoc analyses between each sensory index pair (SOM–VIS, SOM–VES, SOM–VP, VIS–VES, VIS–VP, and VES–VP) [29]. Descriptive statistics, including median (Md) and interquartile range (IQR), were provided for each index.

The Spearman correlation coefficient was used to assess the agreement between each sensory participation index calculated based on sway from the lateral malleolus and mastoid process during m-CTSIB. Additionally, the Spearman correlation coefficient was employed to explore the association between different sensory participation indexes and PBS scores.

The confidence level was set at 95%, with associated *p*-values less than 0.05 considered statistically significant. Statistical analyses were conducted using Jamovi^®^ Software 2.3 (2022) for R [30,31].

## 3. Results

### 3.1. Sample Characterization

Initially, the study included a total of 30 participants, but the final sample comprised 27 children (22 boys and 5 girls) due to difficulties encountered by 2 participants in understanding and executing simple activities based on verbal instructions and imitation, and another participant’s incomplete assessment due to behavioral alteration. Sociodemographic and clinical data are presented in Table 1.

### 3.2. Differences in the Use of Sensory Information in m-CTSIB

Significant differences were observed in the utilization of sensory participation indexes for PC maintenance calculated from the lateral malleolus (*X*^2^ = 23.5; *df* = 3; *p* < 0.001). A greater reliance on somatosensory information compared to visual, vestibular, or visual preference was noted to maintain PC while standing. Minimal body displacement was recorded in the SEC condition from the lateral malleolus, indicating heightened reliance on somatosensory information due to the cancellation of visual input. No significant differences were found between the utilization of visual, vestibular, or visual preference information when compared pairwise.

Similarly, significant differences were noted in indexes calculated from the mastoid process (*X*^2^ = 27.8; *df* = 3; *p* < 0.001). Once again, greater reliance on somatosensory information compared to visual, vestibular, or visual preference was observed. No differences were found between the utilization of vestibular and visual information. However, the utilization of visual preference use was significantly lower than that of vestibular and visual information (Table 2).

When analyzing the agreement between each sensory index calculated from the Kinovea^®^ analysis of anterior and posterior sway of markers placed on the lateral malleolus and mastoid process, we observed a significant positive correlation between each sensory participation index measured at both markers (Table 3).

### 3.3. Pediatric Balance Scale

The overall balance ability of the sample of children with ASD, as measured with the PBS, was 50.44 ± 2.74.

Regarding the association between PBS scores and the various sensory participation indexes of the m-CTSIB, the results revealed a significant negative correlation only with the vestibular index, computed from both markers (lateral malleolus *Rho* = −0.431, *p* = 0.03; mastoid process *Rho* = −0.448, *p* = 0.02).

## 4. Discussion

### 4.1. Sample Characterization

To best of the authors’ knowledge, this study represents the first investigation addressing PC in such a young population with ASD. The selected sample comprised children aged between 3 and 6 years, a critical period for accessing early-stage developmental treatment. Previous studies typically recruited older children and were conducted in different research settings [13,32,33,34]. The primary novelty of this study lies in recruiting children with ASD in an Early Intervention Center, where they typically undergo therapy, thereby maintaining their natural environment. Considering that children with autism often experience heightened anxiety in unfamiliar settings and with unfamiliar individuals, conducting assessments in their natural environment and with familiar assessors can enhance the reliability of their functional capabilities [19]. Furthermore, the mean age of the sample is 4.59 ± 0.69 years, an optimal age for analyzing PC while standing, although previous studies assessed children older than 6 years old [13,32,33,34].

Regarding the characteristics of the sample, the children’s heights and weights fell within the normal ranges described for Spanish children [35]. There was some variability in terms of the age of diagnosis and the onset of sitting and walking, but the mean values were within the upper limits of normality, except for a few children. In terms of motor development, the sample in this study achieved sitting at 8 ± 2.29 months, while TD children usually achieve this milestone at 5.9 months in 50% and at 6.8 months in 90% of cases. Additionally, 50% of TD children usually begin walking at 12.3 months and 90% at 14.9 months [26], whereas the children with ASD in our sample began walking at 15.85 ± 4.6 months. The children with ASD in the sample acquired developmental milestones at later ages compared to TD children, as observed in other studies; this delay could be partially attributed to difficulties in visuo-vestibular and visuo-somatosensory integration [9,26]. Despite this, only four of the participants in the present study attended physiotherapy sessions. There appears to be a lack of access to physical therapy treatments for children with ASD, despite the existing evidence demonstrating motor difficulties associated with this disorder; an early intervention is highly recommended, as early motor interventions may mitigate the negative impact of motor problems on early social communication skills, and the acquisition of new and more complex gross and fine motor abilities allows infants to obtain more information about the social and physical worlds [9].

### 4.2. Differences in Sensory Information Use in m-CTSIB

PC in younger children with ASD has been understudied. Previous research on the use of sensory information for PC in this population is limited. One recent study used the Biodex Balance System to examine postural stability in 38 children aged 6 to 14 with ASD. They also assessed sensory integration and balance with the m-CTSIB. This research found that children with ASD have PC deficits, especially when visual and somatosensory inputs are disrupted, compared to a control group matched for age and sex [13]. However, another recent study did not find difficulties in 14 children aged 7 to 12 with ASD assessed with SOT [11]. The variability in the study results underscores the diversity in PC among children with ASD, a common trait in this population. Notably, all previous studies have included children older than 6, and the effects of visual interferences on PC and their relationship with early development remain unknown.

In our sample, the children with ASD had more difficulty maintaining PC while standing on an unstable surface with closed eyes, indicating a greater reliance on somatosensory information compared to visual or vestibular cues. However, we also observed increased sway associated with the visual preference index, suggesting that children with ASD in our study were more reliant on visual information for maintaining PC when it was naturally present, but that this information was less effective when it was presented variably and required attention [11]. These findings align with a study developed by Cambier et al. in TD children aged 4 to 5, which also showed a preference for somatosensory information and increased sway when standing on an unstable surface with closed eyes [36].

We found significant positive correlations between sensory indexes measured at both markers. However, there were significant differences in vestibular information use when analyzing sway of the mastoid process, especially in the presence of visual interference. This may be because measuring anteroposterior swing of the mastoid process captures the head movements needed to stabilize gaze in conditions with visual interference [37]. Other studies have also found decreased sway in children with ASD when focusing on visual tasks, suggesting differences in attentional demands between variable stimuli and visual recognition tasks, which impact PC differently [33]. The differences observed may be due to the varying attentional demands when children are required to focus on a stimulus that changes compared to when they perform a visual recognition task, as the perceptual and cognitive demands differ. Consequently, their impact on PC is different [38,39]. These findings suggest an important reliance on somatosensory inputs in children with ASD, particularly regarding visual and vestibular information. However, challenges with exclusive use of somatosensory information have also been documented [40].

Regarding difficulties with somatosensory inputs, Stins et al. [7] showed significant differences in PC between nine children with ASD and a mean age of 10.8 years old and controls when using somatosensory inputs. Similar findings were reported in a study by Abdel Ghaffar et al. [13], who studied 78 children with ASD aged 6 to 14 years, calculating visual and somatosensory participation indexes by analyzing postural sway under all four conditions of the m-CTSIB applied on a force platform. Our results indicate the importance of addressing these challenges during the preschool years, a critical period for somatosensory interaction and motor skill development, in which the interaction with peers is mainly achieved though somatosensory games in sensory-challenging environments, such as playgrounds [24,34]. Therefore, appropriate relevance should be given to enhancing their motor skills to improve their participation. Also, Travers et al. [34] pointed out that everyday activities often involve changing surfaces, highlighting the need to evaluate and address these specific challenges in clinical settings.

### 4.3. Pediatric Balance Scale

In our sample of children with ASD, the PBS scores were similar to those of TD children, indicating comparable balance abilities. The mean PBS score was 50.44 ± 2.74 points, falling within the range of reference values for TD between 4 years and 4 years 5 months (49.5 ± 5.76) and between 4 years 6 months and 4 years 11 months (51.2 ± 5.07) [28]. These results suggest that children with ASD perform similarly to their TD peers on the PBS. Notably, the PBS involves vision deprivation in only one task, with the testing surface remaining stable throughout. However, it is noteworthy that the PBS may not fully capture the specific sensory processing challenges encountered by children with ASD, as previous research has indicated normal or near-maximum scores in children with autism older than 6 years [41].

Regarding the relationship between the PBS scores and the different sensory participation indexes of the m-CTSIB, our findings only revealed a significant negative correlation with the vestibular index. This correlation may be explained by the fact that the vestibular system is the last sensory system to reach functional maturity and contribute to PC [42].

### 4.4. Limitations and Future Lines of Research

This study is subject to certain limitations that warrant consideration. The absence of a control group precludes direct comparison of our results with those of age-matched TD children. To better understand the disparities in PC between our sample and TD children, we utilized the PBS as an objective measure, providing normative data for comparison. Future research avenues should include case-control studies to obtain more precise insights into the differences in sensory processing during standing between children with ASD and their TD counterparts. Additionally, incorporating measures of language, social skills, and sensorimotor development could enhance our understanding of the interplay between various aspects of overall development and their impact on the activities and participation of children with ASD [9].

We did not explore the relationship between height, weight, and PC in the children with ASD in our sample, nor did we examine their relationship with developmental milestones, as these were not the primary objectives of our study. Investigating the associations between sociodemographic, clinical, and developmental milestones and the development of PC in children with ASD could be a promising avenue for future research. Such endeavors would necessitate larger sample sizes, case-control studies, and longitudinal designs to gain a deeper understanding of the factors influencing PC development and its correlates.

Furthermore, our administration of the m-CTSIB took place in a natural environment and included two additional conditions, following a methodology similar to that of previous studies [16]. We utilized Kinovea^®^ software, which has not been validated for use in children with ASD, and endeavored to replicate laboratory conditions in a natural setting. While this approach may be less reliable, the correlation between data obtained from two anatomical reference points supports the reliability of our findings. Moreover, conducting assessments in children’s natural environments presents an opportunity for a more holistic evaluation and better tolerance of evaluation procedures. Investigating differences in children’s behaviors across different contexts could be a valuable research direction for gaining insights into the environmental influences on PC and sensory processing. Future studies should address these limitations while leveraging the strengths of our study to develop assessments that comprehensively evaluate children’s functionality in their natural environments, thus minimizing their discomfort and providing more accurate data regarding their limitations in activities and participation.

## 5. Conclusions

Children aged 3 to 6 years with ASD in the analyzed sample, when assessed using the m-CTSIB with Kinovea^®^ software, exhibited notable distinctions in sensory participation indexes for PC maintenance, as derived from lateral malleolus and mastoid process sway. A pronounced reliance on somatosensory inputs over visual or vestibular cues for maintaining PC while standing was evident. However, when comparing all sensory participation indexes in pairs based on lateral malleolus sway, no significant differences were detected between the utilization of visual and vestibular cues. Conversely, analysis of mastoid process sway revealed that the visual preference index significantly exceeded both vestibular and visual indexes. This finding suggests that dependence on visual information may be more effective for PC maintenance when such information is readily available within the assessment environment, as opposed to situations where it is variable and requires cognitive attention.

A significant positive correlation was observed between each sensory participation index measured at both markers. Overall, the children with ASD in the studied cohort demonstrated a preference for utilizing somatosensory inputs, particularly in scenarios where visual information was absent. They did not exhibit discernible differences between the involvement of visual and vestibular cues in maintaining PC while standing, although they experienced greater instability when visual information was presented as interference.

The mean PBS score for the sample of children with ASD was 50.44 ± 2.74, falling within normal ranges. Notably, these results exhibited a significant negative correlation only with the vestibular index, calculated from both markers.

The findings of this study underscore the importance of early and targeted assessment of sensory processing involved in PC within the natural contexts of children. Such assessments could significantly contribute to the identification of PC difficulties that impede the activities and participation of children with ASD.

## Figures and Tables

**Table 1:** Characteristics of the sample.

Variable	Mean ± SD	Maximum	Minimum
Age (years)	4.6 ± 0.7	6	3
Weight (kg)	21.0 ± 4.3	33.3	16.4
Height (m)	1.09 ± 0.07	1.22	0.99
Age of diagnosis (months)	33.2 ± 14.7	65	3
Age of sitting acquisition (months)	8.0 ± 2.3	12	5
Age of gait acquisition (months)	15.8 ± 4.6	30	10

SD: standard deviation.

**Table 2:** Comparisons of sensory participation indexes.

Marker	Index	Median	IQR	Post Hoc Comparisons		
Lateral Malleolus				Comparison	Statistic	*p*-Value
Somatosensorial		0.69	0.88	Som–Vis	3.56	<0.001
Visual		2.11	1.97	Som–Ves	4.05	<0.001
Vestibular		1.96	3.23	Som–VP	5.40	<0.001
Visual preference		4.85	11.7	Vis–Ves	0.49	0.625
				Vis–VP	1.84	0.069
				Ves–VP	1.35	0.181
**Mastoid Process**						
Somatosensorial		0.79	0.85	Som–Vis	3.19	0.002
Visual		1.10	0.89	Som–Ves	2.93	0.004
Vestibular		1.01	1.12	Som–VP	6.38	<0.001
Visual preference		1.89	0.99	Vis–Ves	0.26	0.799
				Vis–VP	3.19	0.002
				Ves–VP	3.44	<0.001

IQR: interquartile range; Som: somatosensorial; Vis: visual; Ves: vestibular; VP: visual preference.

**Table 3:** Correlation coefficients between sensory participation indexes calculated from lateral malleolus and mastoid process markers.

Sensory Participation Index	*Rho*	*p*-Value
Somatosensorial	0.504	0.008
Visual	0.445	0.021
Vestibular	0.495	0.009
Visual preference	0.425	0.028

## Data Availability

The data presented in this study are available on request from the corresponding author, P.M.-C. The data are not publicly available due to privacy or ethical restrictions.

## Supplementary Materials

The following supporting information can be downloaded at https://www.mdpi.com/article/10.3390/children11030303/s1, Video S1, Visual interference.
